# Evaluation of Reference Genes for Quantitative Reverse Transcription Polymerase Chain Reaction in *Bactrocera dorsalis* (Diptera: Tephritidae) Subjected to Various Phytosanitary Treatments

**DOI:** 10.3390/insects12100945

**Published:** 2021-10-18

**Authors:** Yue Cao, Baishu Li, Naizhong Chen, Ding Yang, Li Li, Tao Liu

**Affiliations:** 1 Institute of Equipment Technology, Chinese Academy of Inspection and Quarantine, Beijing 100123, China; SY20203192940@cau.edu.cn (Y.C.); libaishu@163.com (B.L.); Chennz@caiq.org.cn (N.C.); 2 Department of Entomology, China Agricultural University, Beijing 100193, China; dyangcau@126.com

**Keywords:** quantitative reverse transcription polymerase chain reaction, reference gene, *Bactrocera dorsalis*, phytosanitary treatment

## Abstract

**Simple Summary:**

In this study, seven internal reference genes (G6PDH, GAPDH, RPL-32, Rpl-13, Rps-3, α-tub, and 18S) of *Bactrocera* *dorsalis* under different quarantine treatments (heat treatment, cold treatment, methyl bromide fumigation, and irradiation) were screened. Finally, the most stable internal reference gene was selected, which laid a foundation for the further study of its resistance mechanisms to some abiotic stresses.

**Abstract:**

*Bactrocera dorsalis* is a major pest that causes serious damage to many fruits. Although phytosanitary treatment methods have been developed for *Bactrocera* control, there is a lack of information related to the gene expression pattern of *B*. *dorsalis* subjected to phytosanitary treatment conditions. Prior to quantitative reverse transcription polymerase chain reaction analysis of the most stable reference genes in *B. dorsalis* (Diptera: Tephritidae), *B. dorsalis* third-instar larvae were exposed to various phytosanitary treatments; seven candidate reference genes (18S, G6PDH, GAPDH, RPL-13, RPL-32, RPS-3, and α-Tub) were amplified and their expression stabilities were evaluated using geNorm, NormFinder, BestKeeper, and RefFinder algorithms. Different reference genes were found under different stress conditions. G6PDH was the most stable gene after heat treatment. After cold treatment, α-Tub exhibited the highest expression stability. G6PDH expression stability was the highest after fumigation with methyl bromide. RPL-32 showed the highest expression stability after irradiation treatment. Collectively, RefFinder analysis results revealed G6PDH and RPL-32 as the most suitable genes for analyzing phytosanitary treatment in *B. dorsalis*. This study provides an experimental basis for further gene expression analyses in *B. dorsalis* subjected to various phytosanitary treatments, which can aid in the development of novel phytosanitary treatments against insect pests.

## 1. Introduction

The oriental fruit fly, *Bactrocera dorsalis* (Diptera: Tephritidae) (Hendel), is considered an important agricultural pest that causes serious damage to many fruits [1,2,3]. Female adults lay eggs inside the fruits; the larvae feed from the fruit until pupation, which affects fruit yield [4]. As these larvae have the ability to hide in fruits, *B. dorsalis* has spread to several countries, owing to the international fruit trade, and are considered major quarantine pests in many countries, including the USA, Australia, Japan, and the EU [5]. Therefore, it is necessary to perform phytosanitary treatments on fruits before export [6].

At present, many phytosanitary treatment methods have been developed for the control of *B. dorsalis*. For example, irradiation with a minimal dose of 87.72 Gy potentially results in a sterility rate of 99.9968% [7]. Cold treatment for 15 days at 1.7 °C provides quarantine security for controlling *B. dorsalis* at an efficacy level of 99.9916% [8]. Heat treatment with a fruit core temperature above 46 °C can be used to disinfest *B. dorsalis* [9]. Fumigation with 32 g/m^3^ of methyl bromide (MB) for 4 h has the potential of completely controlling *B. dorsalis* [10]. Despite the available interventions for controlling *B. dorsalis*, there exist major challenges, including damage caused to the host fruit by the treatment doses used for pest control. For example, some varieties of citrus fruits are not tolerant to irradiation [11]. Cold treatment causes serious injury to many tropical fruits, such as banana and pineapple [12,13]. Heat treatment requires raising the core temperature of the fruit to 46–49 °C, and only a small number of tropical fruits, such as papayas and mangoes, can tolerate such high temperatures [10,14]. MB (32 g/m^3^ for 2 h at 20 °C) causes severe damage to mandarin and loquat fruit [15,16]. Thus, there is a need to further develop existing treatment methods, and, as the study of molecular response mechanism accelerates the development of new pesticides [17,18], it is necessary to study the molecular response mechanism of *B. dorsalis* under different treatments.

Gene expression analysis is a widely used and powerful method for studying gene function and metabolic pathways in organisms under biotic and abiotic stresses [19]. One of the most important methods to study gene expression is quantitative real-time polymerase chain reaction (RT-qPCR), which has many advantages, including accuracy, repeatability, high sensitivity, high throughput, and easy operation [20]. When this technique is used to quantify the relative differential expression of genes of interest, the expression value of these genes is compared with the value of genes that are stably expressed among treatments (usually known as control, reference, calibrators, normalizers, or housekeeping genes) and that are used to normalize the differences among samples due to uncontrolled factors that serve as sources of variation. Therefore, comparison with reference genes helps to accurately quantify the expression of genes of interest between different treatments [21,22]. Therefore, the correct selection of internal reference genes determines the accuracy of RT-qPCR to a certain extent. The qPCR uses a cyclic threshold (CT) to define the level of gene expression. It is defined as the number of cycles that the fluorescent signal in each reaction tube goes through when it reaches a set threshold, which is proportional to the number of initial templates present in the reaction. In the case of amplification with intercalating agents, the specificity of the amplification product is detected by evaluating its melting temperature (Tm), which corresponds to the temperature at which 50% of the copies of that sequence present in a reaction are in single-stranded form and 50% are in double-stranded form. This method can be used to determine the specificity of the response. Because the sequence of each amplification is different, each amplification has a unique Tm; so, it is expected that for a specific qPCR reaction with an intercalating agent, there will be a single product and a single Tm.

To date, only a few studies have screened and analyzed reference genes of important agricultural insects subjected to different phytosanitary treatments. For example, the gene expression stability of *B. dorsalis* in different tissues has been studied [23]. Reference genes in *B. cucurbitae* (Coquillett) have been selected under temperature stress [19], and reference genes of body parts, developmental stages, and endogenous genes in the reproductive and olfactory tissues of the medfly and olive fly have been studied [24]. However, there is a lack of research on the gene expression stability of *B. dorsalis* under different phytosanitary treatment conditions. Therefore, in this study, seven candidate internal reference genes, GAPDH, G6PDH, 18S, RPL-13, RPL-32, RPS-3, and α-Tub, of *B. dorsalis* were studied under different phytosanitary treatment conditions using RT-qPCR and three analytic software packages, geNorm [25], NormFinder [26], and BestKeeper [27]. To eliminate the bias of a single evaluation software, an online reference gene evaluation software, RefFinder, was used [28]. Collectively, the findings of our study provide the most suitable reference gene for future research on phytosanitary treatment of *B. dorsalis.*

## 2. Materials and Methods

### 2.1. Insect Rearing

A *B. dorsalis* colony was collected from an insect-infested guava orchard in Guangdong province, China, on 20 September 2014, reared for phytosanitary treatment in the laboratory of the Administration of Quality Supervision, Inspection and Quarantine [15], and was rejuvenated with field-collected fruit flies every 9–12 months [29]. The colony was reared at 26 ± 1 °C and 60 ± 5% relative humidity (RH) with a photoperiod of 12:12 h (dark:light) [6]. Eggs were collected from the colony and incubated to the third-instar larval stage on an artificial diet [15,30].

### 2.2. Candidate Reference Genes

Seven housekeeping genes were selected as candidate reference genes, including α-Tub, GAPDH, 18S, G6PDH, RPS-3, RPL-13, and RPL-32. Primer sequences of the genes used for RT-qPCR analysis and amplification efficiency of each primer are shown in Table 1. To confirm the gDNA was removed by reverse transcription, we amplified a region of the G6PDH gene, which is 609 bp with introns (gDNA). Meanwhile, the gDNA of *B. dorsalis* was used as positive control (Supplementary Figure S1).

In order to get the amplification efficiency of each primer, we used the standard curve method. (1) The reverse transcription cDNA was diluted by 10-fold gradient and then set to S1–S6 with six gradients. (2). A 20-μL qPCR reaction system was prepared. (3) Three technical repetitions were set for each sample. (4) The obtained data were plotted as a standard curve, with the log value of the dilution multiple of the template series as the x axis and the corresponding CT value as the y axis; the reaction efficiency of each primer set was estimated with the following equation: amplification efficiency = [10(−1/slope)]−1.

### 2.3. Phytosanitary Treatments of B. dorsalis

Sixty (60) third-instar larvae of *B. dorsalis* were placed in a cylindrical box (6 cm in diameter and 4 cm in height) with a circular hole (1.5 cm in diameter) at the top. Irradiation, fumigation, cold treatment, and heat treatment were performed, with three replicates for each treatment; a blank control group was set up without treatment. After these treatments, four larvae were selected, washed with 5 mL of water for 1 min, and carefully wiped with absorbent paper to ensure no residual water stains. The larvae were then placed in a 1.5-mL vial, frozen with liquid nitrogen, and stored at −80 °C until further use. The mortality of each treatment was calculated, except for the irradiation group. For heat treatment, cold treatment, and MB fumigation, third-instar larvae were maintained for 2 d at rearing temperature. Larvae not responding when prodded with a blunt probe were recorded as dead, and mortality was defined as the number of unresponsive larvae divided by the total. For irradiation, larvae were transferred to moist sand [7] that had been previously sprayed with a watering can for pupation and adult emergence. Larvae that did not successfully emerge were considered sterile, and the sterility rate was defined as the number of larvae that failed to emerge divided by the total number of larvae.

When the larvae treated with irradiation metamorphosed into insects (flies), they were selected and placed on wet sandy soil until they pupated and then we calculated the sterility rate.

#### 2.3.1. Heat Treatment

The larvae were subjected to heat treatment in an environment-controlled chamber (KBF720, WTC Binder, Tuttlingen, Germany). The following heating program was used: The temperature was raised from 25 °C to 44.5 °C in 1 h at 50% RH and then raised from 44.5 °C to 47.5 °C in 1 h at 95 % RH [9]. A batch of insects was collected when the temperature reached 47.5 °C, and another batch was collected after 2 min.

#### 2.3.2. Cold Treatment

The larvae were subjected to cold treatment in an environment-controlled chamber (KBF720, WTC Binder, Germany). The cooling program was set up as follows: The temperature was maintained at 25 °C for 5 min, reduced from 25 °C to 5 °C in 5 h, reduced again from 5 °C to 1 °C in 5 h, and then maintained at 1 °C [8]. Two groups of larvae were removed after treatment at 1 °C for 18 h and 30 h, respectively.

#### 2.3.3. Irradiation Treatment

The larvae were irradiated using an RS-2000 Pro X-ray irradiator (Rad Source Technologies, Inc., Coral Springs, FL, USA) with operating parameters of 220 KV and 17.6 mA, as previously described by Zhan et al. (2020) [33]. Two groups of larvae were irradiated at doses of 40 Gy and 80 Gy, respectively.

#### 2.3.4. MB Fumigation

Cylinderlized MB (25 kg) with 99.5% purity was purchased from Lian yun gang Dead Sea Bromine Company Ltd. (Lianyungang, China) [6]. Fumigation tests were performed in modified 6-L glass desiccators equipped with small fans, as previously described by Liu et al. (2020) [34]. Two groups of insects were treated with 6 and 7.5 g/m^3^ of MB, respectively, for 3 h at 25 °C [35].

### 2.4. Total RNA Extraction and cDNA Synthesis

Total RNA was extracted from insect samples using the RNA simple Total RNA Kit (Tiangen, Beijing, China). Total RNA integrity was confirmed using 1% agarose gel electrophoresis. Total RNA concentration and quality were evaluated using a spectrophotometer (Nano Drop 2000, Thermo Fisher, Waltham, MA, USA). Each sample (2.0 μg RNA) was reverse transcribed with random primers using the FastQuant RT Kit (with gDNase) (Tiangen) in one batch and then stored at −80 °C for about 1 week until further analysis.

### 2.5. RT-qPCR

RT-qPCRs were performed using Applied Biosystems ViiA™ 7 Real-Time PCR (Thermo Fisher). The reaction mixture for RT-qPCR comprised a total volume of 20 μL consisting of the following: 10 μL of 2 × *Perfect Start*^TM^ Green RT-qPCR SuperMix + DyeII, 0.4 μL each of F/R (Forward and Reverse primers), 1 μL of cDNA template, and 8.2 μL of sterile, double-distilled water. The cycling program comprised an initial denaturation of 10 min at 95 °C, followed by 40 cycles of denaturation at 95 °C for 15 s, annealing for 30 s at 58 °C, and extension for 32 s at 72 °C. After the reaction, a melting curve analysis from 60 °C to 95 °C was applied to all reactions to ensure consistency and specificity of the amplified products.

### 2.6. Data Analysis

Mortality and sterility rates of larvae were calculated using Microsoft Excel 2007. The expression stability of candidate internal reference genes in different samples was analyzed using geNorm, NormFinder, and BestKeeper using the Ct values as input. We used RefFinder to integrate the results of geNorm, NormFinder, and BestKeeper and selected the most stable genes under the tested conditions, as RefFinder software provides a final comprehensive ranking of the stability of the reference genes based on the abovementioned programs [31]. The geNorm program was also used to calculate the optimal number of reference genes required for accurate normalization based on pairwise variation analysis, where V*_n_*_/(*n*+1)_ stands for paired variation and M stands for average expression stability. Based on the analysis of variance, NormFinder evaluated the expression stability of the original Ct values of the candidate internal reference genes after 2^−Δt^ conversion. The lower the calculated stability values, the more stable the gene expression.

## 3. Results

### 3.1. Response of B. dorsalis to Various Phytosanitary Treatments

As shown in Table 2, the mortality rates of third-instar larvae of *B. dorsalis* were between 1.5% and 5% after cold and heat treatments and MB fumigation at low doses. The mortality rates were markedly increased to a range between 7% and 11% following high-dose MB treatment. The sterility rate of irradiated larvae reached 90% at low doses and 98% at high doses. In the control group, the mortality rate was 0%.

### 3.2. Analysis of Total RNA Quality, Primer Specificity, and Expression Stability of Reference Genes under Different Phytosanitary Treatments

Total RNA concentration ranged from 1396 ng/μL to 2780 ng/μL, and RNA purity (A260:A280) was high, with values ranging from 2.14 to 2.46 (Table S1). Using Shen’s method [23], we confirmed the complete removal of genomic DNA from the RNA samples (Figure S1).

**Figure 1:**
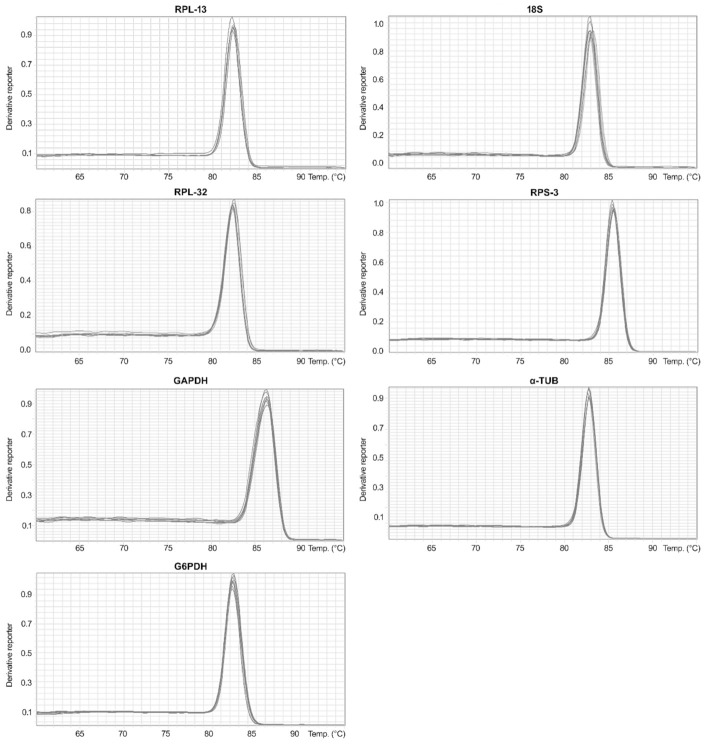
Melting curves for the seven candidate reference genes. The x axis is the temperature and the y axis is the Derivative Reporter.

Seven candidate reference genes in all samples exhibited melting curves with a single peak, indicating that the primers were highly specific (Figure 1). Tm values of the seven genes are shown in Table 3.

**Figure 2:**
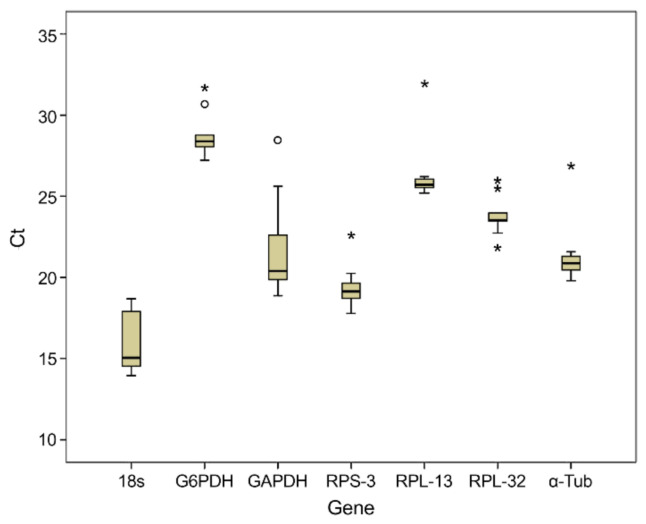
Ct values for all candidate reference genes in *B. dorsalis* under various phytosanitary treatment conditions. The x axis is gene, y axis is the Ct value, “○” is a mild outlier, and “*” is an extreme outlier. The two horizontal lines above and below each box represent the maximum and minimum values of Ct, the middle horizontal line represents the median, and the upper and lower horizontal lines of the box represent the upper and lower quartiles, respectively.

Figure 2 shows an analysis of the expression pattern of all tested reference genes in all samples, identifying variations between the reference genes. Each biology was repeated three times. The Ct (Cycle threshold) values of the seven genes ranged from 13.96 to 31.95, and the specific scope is shown in Table 4. Compared with other genes, the expression of 18S gene was high, reaching the threshold fluorescence after 13.96 amplification cycles. Contrastingly, the average Ct value of all reference genes in the data set was approximately 23 cycles. The expression range of the seven reference genes was very wide, indicating the importance of selecting reliable reference genes for regulating gene expression under certain conditions.

### 3.3. The geNorm Analysis

**Figure 3:**
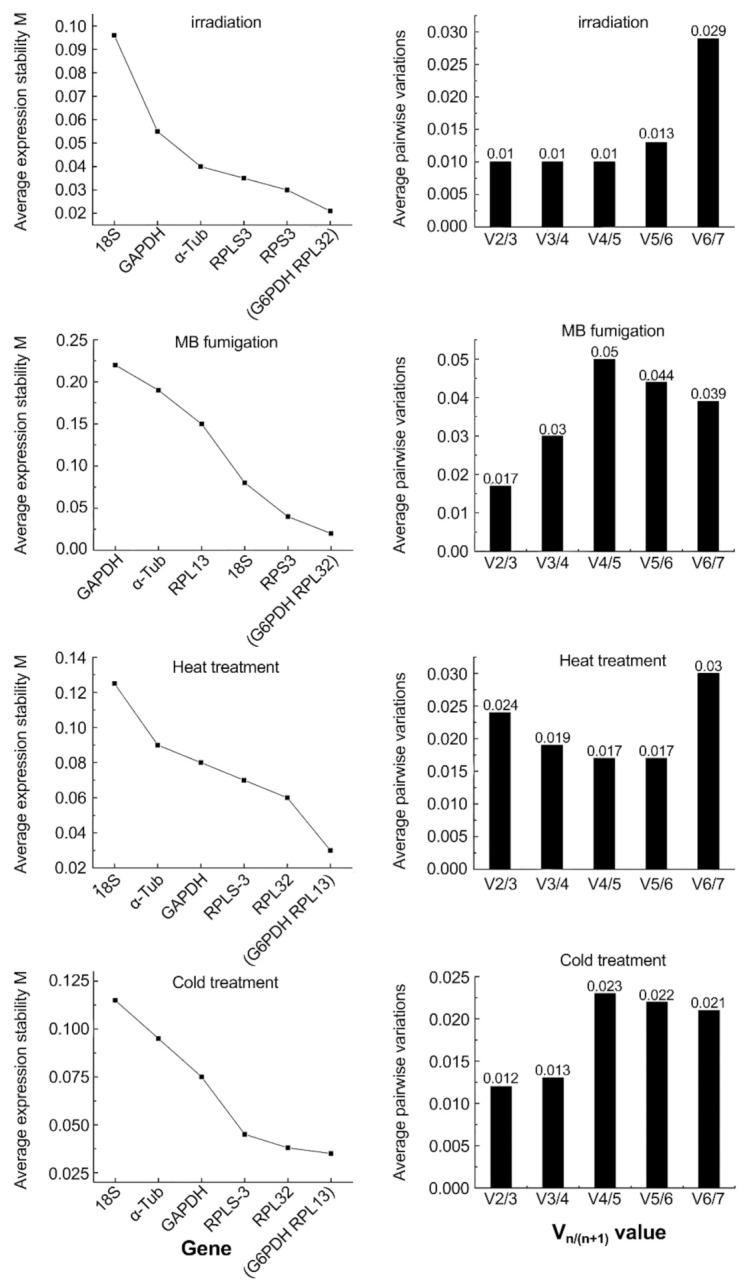
Analysis of expression stability and evaluation of optimal number of reference genes in *B. dorsalis* under different phytosanitary treatment conditions using geNorm. The left graph’s y axis is average expression stability M, and the x axis is genetic stability, increasing from left to right. The right graph’s y axis is average pairwise variations, and the x axis is V*_n_*_/(*n*+1)_ values).

The stability of internal reference genes was analyzed by geNorm (Figure 3). Under different phytosanitary treatment conditions, the stability of the reference genes was different. G6PDH and RPL-13 were the most stable genes in heat treatment, G6PDH and α-Tub were the most stable genes in cold treatment, G6PDH and RPL-32 were the most stable genes in fumigation, and G6PDH and RPL-32 were the most stable genes in irradiation. G6PDH was the gene that was stable in the greatest number of treatments. Moreover, results from low- and high-dose MB treatments were consistent.

The geNorm was used to analyze pairwise variation (using V*_n_*_/(*n*+1)_ values) to determine the optimum number of internal reference genes. When the V*_n_*_/(*n*+1)_ value is less than 0.15, the *n* + 1 gene need not be introduced for correction, and the most suitable number of internal reference genes is *n*. In contrast, a new gene correction is introduced until V*_n_*_/(*n*+1)_ is less than 0.15. The V*_n_*_/(*n*+1)_ values obtained in the present study are shown in Figure 3. These results showed that a third reference gene was not needed for gene expression analysis under different phytosanitary treatment conditions, and the most suitable number of internal reference gene combinations was two.

### 3.4. NormFinder Analysis

The most stable genes of *B. dorsalis* from the third-instar larvae differed among the four phytosanitary treatments. GAPDH and RPL-13 were the most stable genes in heat treatment, α-Tub was the most stable gene in cold treatment, GAPDH was the most stable gene in fumigation treatment, and RPL-32 was the most stable gene in irradiation treatment. Moreover, results from low- and high-dose MB treatments were consistent (Table 5).

The stability of the reference genes was determined using BestKeeper, according to the standard deviation (SD) and coefficient of variation (CV) values. The smaller the SD and CV values, the more stable the gene expression. However, when candidate gene SD (±Ct) value was greater than 1, it was considered unsuitable as a reference gene. The results of BestKeeper analysis (Table 6) showed that the stability of reference gene expression differed under diverse phytosanitary treatment conditions; however, the results of low- and high-dose treatments were consistent.

### 3.5. RefFinder Analysis

In the third-instar larvae of *B. dorsalis* treated with heat, the stability of the seven candidate genes showed the following order: G6PDH > RPL-13 > RPL-32 > RPS-3 > α-Tub > GAPDH > 18S. In the third-instar larvae of *B. dorsalis* subjected to cold treatment, the stability of the seven candidate genes showed the following order: α-Tub > RPL-13 > RPL-32 > G6PDH > 18S > RPS-3 > GAPDH. In the third-instar larvae of *B. dorsalis* subjected to fumigation, the stability of the seven candidate genes showed the following order: G6PDH > RPL-32 > RPS-3 > RPL-13 > 18S > α-Tub > GAPDH. In the third-instar larvae of *B. dorsalis* treated by irradiation, the stability of the seven candidate genes showed the following order: RPL-32 > RPS-3 > RPL-13 > G6PDH > α-Tub > GAPDH > 18S. Collectively, these results showed that the final stability of the reference genes of the third-instar larvae of *B. dorsalis* was as follows: G6PDH = RPL-32 > RPS-3 > RPL-13 > α-Tub > 18S > GAPDH.

## 4. Discussion

Many species belonging to the family Tephritidae, including *B. dorsalis* and *B. minax*, are pests that largely affect international trade; therefore, phytosanitary treatments are crucial for controlling the spread of these pests. *B.* *dorsalis* has been widely studied as an important quarantine pest. Although a few studies have screened and analyzed reference genes under abiotic stress, no studies have reported the reference genes under quarantine treatment. Therefore, in this study, we selected four different quarantine treatment conditions and analyzed the expression patterns of selected reference genes in *B. dorsalis* under these treatments.

Based on the results of previous studies, we screened seven genes (G6PDH, 18S, GAPDH, RPL-32, RPL-13, RPS-3, and α-Tub) and analyzed them under different phytosanitary treatment conditions. According to our results, no selected gene was suitable as a universal reference gene under the four treatments, which could be attributed to the different cell functions under abiotic stress, and these findings were consistent with those of previous studies [36]. We found that the 18S gene was unstable under the four quarantine treatments, although a previous study showed this reference gene as being the most stable in *B. cucurbitae* (Coquillett) following heat treatment [19]. These observations could be attributed to the different temperatures used in the two experiments. We also found that RPL-32 was relatively stable during heat, cold, and irradiation treatments as well as during fumigation, which was consistent with the findings of a previous study on different developmental stages of *B. minax* [31].

In addition, we found that α-Tub was stable only in the cold treatment and unstable in the other three treatments. Shen (2010) reported that α-Tub expression is relatively stable in different tissues of *B. dorsalis*, which could be linked to the abiotic stress response mechanisms [23]. At present, there is no explanation available for the contrasting observations and, therefore, further studies are required to explore this phenomenon. Although GAPDH is often used as a standardized endogenous control in different tissues of *B. dorsalis* and *B. minax* [37], GAPDH was the most unstable in our results. This could be attributed to the same reason stated above for the 18S gene; however, further research is needed.

To increase the reliability of the experimental results, we used three different algorithms (geNorm, NormFinder, and BestKeeper) to select the reference gene. Of all genes, G6PDH, RPL-32, RPL-13, and α-Tub ranked the highest in geNorm and NormFinder, and G6PDH, RPL-13, and RPL-32 ranked the highest in BestKeeper. The rankings assigned by the three different software differed, which has also been noted in previous studies [36,38]. Therefore, it was reasonable to calculate the stability of gene expression using different programs based on different mathematical methods; hence, RefFinder was used to compare the rankings obtained from the three software packages, and the results thus obtained were considered the final ranking [28]. For heat and fumigation treatments, the RefFinder statistical results showed G6PDH as the most suitable reference gene for all treatments. The α-Tub was considered the most suitable for cold treatment, whereas RPL-32 was regarded the most suitable for irradiation. These results were consistent with those of previous studies [23,31].

Moreover, in our experiment, all the larvae were fed and treated an artificial diet to make it easier to obtain samples. Although in practice the larvae generally live in fruit, many studies have used artificial diet. Example of this is the paper published in 2010 by Shen [23] on different tissues of *B. dorsalis* treated with an artificial diet, which is also a good support for our experimental results. Meanwhile, we used a sublethal mortality rate; otherwise, the mortality rate would have been too high for genetic analysis. Our results showed that the different doses used in all treatment had no effect on the stability of the reference genes. However, as only two gradients were set due to the different treatments, it is unclear whether the changes in dose and reference gene expression stability are related, and further experiments are needed to verify this.

Several studies have emphasized the use of multiple reference genes to standardize gene expression [39,40]. In the current study, geNorm was used to calculate the optimal number of reference genes required for normalization. Based on the results of this program, all samples were finally ranked by RefFinder; a different gene as an internal normalization control in each treatment should have been used.

## 5. Conclusions

In this study, seven candidate reference genes were selected and their expression stability under four quarantine treatments (heat, cold, MB fumigation, and irradiation) was evaluated using geNorm, NormFinder, BestKeeper, and RefFinder algorithms. The results showed that G6PDH and RPL-13 were the most stable reference genes under heat treatment, α-Tub and RPL-13 were the most stable under cold treatment, G6PDH and RPL-32 were the most stable under MB fumigation, and RPL-32 and RPS-3 were the most stable under irradiation. Our results highlighted the wide expression range of the reference genes used in this study. Furthermore, this study may have potential implications for gene expression analysis of *B. dorsalis* in the future. 

## Figures and Tables

**Table 1:** Primer sequences for seven reference genes used in the RT-qPCR analysis (R^2^, regression coefficient; F, forward primer; R, reverse primer).

Gene Symbol	Gene Name	Primer Sequence (5′ 3′)	GenBank Accession Number	Fragment Length (bp)	Efficiency (%)	R^2^	Reference
18S	18Sr-RNA	F: GCGAGAGGTGAAATTCTTGG	AF033944	191	94.9	0.9962	Shen et al., 2010 [23]
		R: CGGGTAAGCGACTGAGAGAG					
RPL-32	Ribosomal Protein L32	F: CGATTTCTCCGCAGTATTCAC	——	147	107.5	0.9813	Lü et al., 2014 [31]
		R: GCCAGTACCTCATGCCTAACA					
RPL-13	Ribosomal Protein L13	F: CAGTTGTACGTTGCGAGGAATT	HM236866	134	106.7	0.9816	Shen et al., 2012
		R: TCTTGATGGAGCACGGGAG					
GAPDH	Glyceraldehyde-3-phosphate dehydrogenase	F: GACGCCTACAAGCCTGACAT	GU269901	221	90.5	0.9896	Shen et al., 2010
		R: GTTGAAGCGGGAATGATGTT					
G6PDH	Glucose 6-phosphatedehydrogenase	F: CCTACAAACTTCTGCGGTTATGC	AB021910	382	89.1	0.9853	Shen et al., 2010
		R: AGAGCGAGGCGAGGTGATC					
RPS-3	Ribosomal protein S3	F: TGGATCACCAGAGTGGATCA	——	169	99.5	0.9984	Li et al., 2019 [32]
		R: TAAGTTGACCGGAGGTTTGG					
α-Tub	α-Tubulin	F: CGCATTCATGGTTGATAACG	GU269902	184	108.9	0.9742	Shen et al., 2010
		R: GGGCACCAAGTTAGTCTGGA					

**Table 2:** Mortality and sterility rate of *B. dorsalis* third-instar larvae after different phytosanitary treatments.

Treatment Conditions		Mortality Rate (%)			Mean ± SEM
Heat treatment	47.5 °C 0 min	2.1	3.4	1.3	2.27 ± 0.61
	47.5 °C 2 min	7.8	6.4	9.7	7.97 ± 0.96
Cold treatment	1 °C 18 h	4.6	5.8	2.3	4.23 ± 1.03
	1 °C 30 h	10.7	12.4	8.6	10.57 ± 1.1
MB fumigation	6 g/m^3^	1.5	2.3	1.4	1.73 ± 0.28
	7.5 g/m^3^	5.7	6.9	7.9	6.83 ± 0.64
CK (control check)	——	0	0	0	——
	Sterility rate (%)				Mean ± SEM
Irradiation	40 Gy	88	92.3	91.7	90.67 ± 1.34
	80 Gy	99.4	98	98.8	98.73 ± 0.41

**Table 3:** Tm values of seven genes.

Gene	Tm Value ± SEM
18S	83.03 ± 0.27
G6PDH	82.51 ± 0.33
GAPDH	86.29 ± 0.10
RPL-13	82.29 ± 0.20
RPS-3	85.41 ± 0.09
RPL-32	82.39 ± 0.10
α-Tub	82.78 ± 0.10

**Table 4:** The Ct (Cycle threshold) values of the seven genes.

Gene	Minimum Value ± SEM	Maximum Value ± SEM
18S	13.96 ± 0.71	18.68 ± 1.33
G6PDH	18.88 ± 0.62	28.46 ± 0.97
GAPDH	27.21 ± 0.65	31.70 ± 1.67
RPL-13	25.18 ± 0.54	31.97 ± 0.77
RPS-3	17.77 ± 0.31	22.59 ± 2.33
RPL-32	21.84 ± 0.46	25.97 ± 1.27
α-Tub	19.79 ± 1.22	26.88 ± 2.67

**Table 5:** Gene expression stability of tested reference genes of *B. dorsalis* exposed to different phytosanitary treatments analyzed using NormFinder.

Genes	Heat Treatment		Cold Treatment		Fumigation		Irradiation	
	Stability and Ranking		Stability and Ranking		Stability and Ranking		Stability and Ranking	
RPL-13	0.010	1	0.017	2	0.081	3	0.032	4
RPL-32	0.045	2	0.046	4	0.078	2	0.006	1
RPS-3	0.065	4	0.070	5	0.100	4	0.008	2
α-Tub	0.052	3	0.011	1	0.138	5	0.044	6
GAPDH	0.010	1	0.031	3	0.059	1	0.037	5
G6PDH	0.073	5	0.099	7	0.186	7	0.030	3
18S	0.142	6	0.090	6	0.156	6	0.139	7

**Table 6:** Gene expression stability of tested reference genes of *B. dorsalis* exposed to different phytosanitary treatments analyzed using BestKeeper.

Heat Treatment	RPL-13	RPL-32	RPS-3	18S	G6PDH	GAPDH	α-Tub
geo Mean [Ct]	15.83	21.10	28.32	20.89	25.68	23.07	18.89
ar Mean [Ct]	15.92	21.13	28.32	20.91	25.69	23.09	18.91
min [Ct]	13.96	19.88	28.05	19.84	25.46	21.84	17.77
max [Ct]	17.91	22.60	28.51	21.58	26.05	23.97	20.26
stddev [±Ct]	1.33	0.98	0.18	0.71	0.24	0.84	0.90
CV [% Ct]	8.33	4.64	0.63	3.40	0.95	3.62	4.76
Stability rank	2	4	6	7	1	5	3
Cold treatment	RPL-13	RPL-32	RPS-3	18S	G6PDH	GAPDH	α-Tub
geo Mean [Ct]	15.14	22.20	30.26	20.52	25.71	25.16	20.46
ar Mean [Ct]	15.15	22.31	30.29	20.53	25.71	25.17	20.52
min [Ct]	14.53	20.40	28.51	19.84	25.54	23.97	18.71
max [Ct]	15.88	25.62	31.70	21.22	26.06	25.98	22.59
stddev [±Ct]	0.49	2.20	1.19	0.46	0.23	0.81	1.38
CV [% Ct]	3.23	9.88	3.93	2.24	0.90	3.20	6.72
Stability rank	1	4	6	3	5	7	2
Fumigation	RPL-13	RPL-32	RPS-3	18S	G6PDH	GAPDH	α-Tub
geo Mean [Ct]	16.22	22.40	28.37	20.30	25.40	23.67	19.72
ar Mean [Ct]	16.27	22.75	28.37	20.31	25.40	23.67	19.72
min [Ct]	14.93	18.88	27.84	19.79	25.18	23.49	19.26
max [Ct]	18.01	28.46	28.77	21.31	25.54	23.97	20.26
stddev [±Ct]	1.16	3.80	0.36	0.66	0.14	0.20	0.36
CV [% Ct]	7.12	16.72	1.25	3.26	0.56	0.84	1.82
Stability rank	1	2	4	6	3	7	5
Irradiation	RPL-13	RPL-32	RPS-3	18S	G6PDH	GAPDH	α-Tub
geo Mean [Ct]	16.12	20.06	27.92	20.39	25.82	23.67	19.72
ar Mean [Ct]	16.23	20.08	27.92	20.39	25.82	23.67	19.72
min [Ct]	14.13	18.94	27.22	19.84	25.54	23.49	19.26
max [Ct]	18.68	20.92	28.51	20.88	26.21	23.97	20.26
stddev [±Ct]	1.63	0.76	0.47	0.37	0.26	0.20	0.36
CV [% Ct]	10.05	3.80	1.69	1.80	1.00	0.84	1.82
Stability rank	2	1	5	7	4	6	3

## Data Availability

All data presented in this study are available in the article.

## Supplementary Materials

The following are available online at https://www.mdpi.com/article/10.3390/insects12100945/s1, Figure S1. The electrophoregram of PCR of G6PDH gene using different templates. Table S1. Total RNA concentrations and A260: A280 values.
