# Supplementary material for: Evaluation of Reference Genes for Quantitative Reverse Transcription Polymerase Chain Reaction in Bactrocera dorsalis (Diptera: Tephritidae) Subjected to Various Phytosanitary Treatments
Source: Insects. 2021 Oct 18;12(10):945. doi: 10.3390/insects12100945 (PMC8537244; doi:10.3390/insects12100945)
Supplement: Supplementary file 1 [file insects-12-00945-s001.zip › insects-1339558-supplementary.pdf]

Supplementary Materials

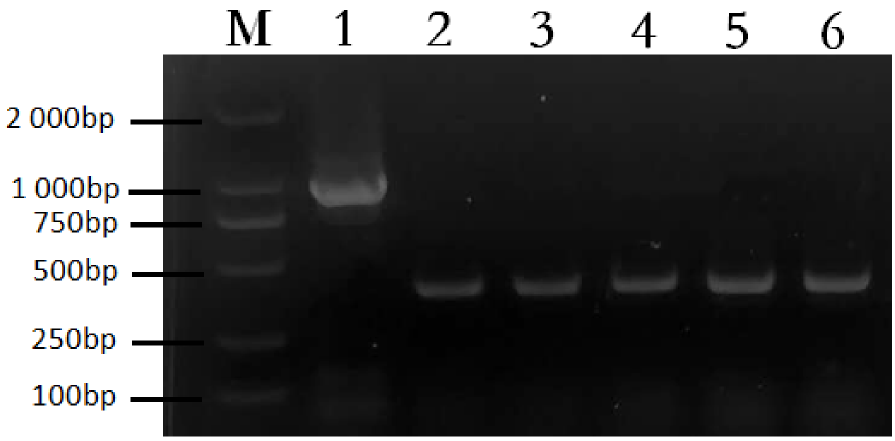

Figure S1. The electrophoregram of PCR of G6PDH gene using different templates. The M lane represents the DNA marker; the 1 lane represents the positive control; and the 2–6 lane represents the expunged cDNA, each sample is mixed with a different dose of the samples.

Table S1. Total RNA concentrations and A260: A280 values. FZ: irradiation; XZ: MB fumigation; R: heat treatment; L: cold treatment. Numbers 1 and 2 represent different doses.

Template RNA	Concentration (ng/μL)	A260: A280
CK	2780	2.17
FZ-1	2106	2.14
FZ-2	2351	2.11
XZ-1	2621	2.19
XZ-2	1842	2.14
R-1	2212	2.20
R-2	1396	2.16
L-1	2040	2.17
L-2	2005	2.22
